# Sono-Elastography: An Ultrasound Quantitative Non-Invasive Measurement to Guide Bacterial Pneumonia Diagnosis in Children

**DOI:** 10.3390/children10081335

**Published:** 2023-08-01

**Authors:** Sergi Huerta-Calpe, Bárbara Salas, Emilio J. Inarejos Clemente, Carmina Guitart, Mònica Balaguer, Iolanda Jordan

**Affiliations:** 1 Pediatric Intensive Care Unit, Hospital Sant Joan de Déu, 08950 Barcelona, Spain; sergi.huerta@sjd.es (S.H.-C.); carmina.guitart@sjd.es (C.G.); monica.balaguer@sjd.es (M.B.); 2 Immune and Respiratory Dysfunction Research Group, Institut de Recerca Sant Joan de Déu, 08950 Barcelona, Spain; 3 Radiology and Diagnostic Imaging Unit, Hospital Sant Joan de Déu, 08950 Barcelona, Spain; barbara.salas@sjd.es (B.S.); emili.inarejos@sjd.es (E.J.I.C.)

**Keywords:** pneumonia, ultrasound, sono-elastography, strain imaging, shear wave imaging

## Abstract

Lung ultrasound (LUS) is, at present, a standard technique for the diagnosis of acute lower respiratory tract infections (ALRTI) and other lung pathologies. Its protocolised use has replaced chest radiography and has led to a drastic reduction in radiation exposure in children. Despite its undeniable usefulness, there are situations in which certain quantitative measurements could provide additional data to differentiate the etiology of some pulmonary processes and thus adapt the treatment. Our research group hypothesises that several lung processes such pneumonia may lead to altered lung tissue stiffness, which could be quantified with new diagnostic tests such as lung sono-elastography (SE). An exhaustive review of the literature has been carried out, concluding that the role of SE for the study of pulmonary processes is currently scarce and poorly studied, particularly in pediatrics. The aim of this review is to provide an overview of the technical aspects of SE and to explore its potential usefulness as a non-invasive diagnostic technique for ALRTI in children by implementing an institutional image acquisition protocol.

## 1. Introduction

Acute lower respiratory tract infections (ALRTI) are highly prevalent in pediatric patients and represent one of the main reasons for hospital admissions. Pneumonia, which is included in ALRTI, is the leading cause of death in children worldwide, accounting for more than 2 million deaths each year. It is also the most common cause of childhood morbidity and mortality [1,2] and one of the most common causes of admission to pediatric intensive care units [3]. Clinical manifestations of pneumonia depend on the host, pathogen and severity. As described in the Centers for Disease Control (CDC) guidelines, pneumonia can be the cause of fever, cough, tachypnea, respiratory distress and/or decreased oxygen saturation [4]. However, its clinical signs and symptoms are non-specific and subtle, especially in infants and younger patients who may present with refusal to feed and irritability as the only symptoms of pneumonia. Children and adolescents often present with pleuritic chest pain that characteristically increases with respiration. Abdominal pain, referred to as lower body pain, or nuchal rigidity, referred to as upper body pain, may also be clinical signs of pneumonia [5,6]. The etiology may be bacterial, viral or fungal [7]. Differentiating between bacterial pneumonia (BP) and other causes of respiratory failure with lung consolidation findings such as viral pneumonia (VP), atelectasis or pulmonary contusion can be difficult as the symptoms and clinical signs can be similar [8,9,10]. However, their differentiation is particularly important as an accurate diagnosis will lead to a correct isolation protocol, an appropriate therapeutic approach (including antibiotic prescription and duration) and a reliable prognosis. The diagnosis is, therefore, based on a compendium of clinical findings, the interpretation of certain biochemical parameters (such as leukocytes, neutrophils, C-reactive protein or procalcitonin), microbiological cultures (respiratory samples) and complementary imaging examinations [11,12]. 

Over the last few decades, several imaging tests have been developed to improve the diagnosis of ALRTI. Thus, lung ultrasound (LUS) has been consolidated as an effective technique with high sensitivity and specificity for the diagnosis of these lung processes, with clear advantages over conventional X-ray imaging in terms of reducing radiation exposure and providing close monitoring of the evolution [12,13,14]. However, no pathognomonic or specific findings have been described for its accurate differential diagnosis. In our experience, we believe that the main disadvantage of using LUS in isolation is that it is a subjective test. Although it has high interobserver agreement (our bibliography is attached), it provides non-quantitative measures that sometimes do not allow differentiation between different lung condensations or different interstitial patterns. We have seen that this differentiation could be improved by adding another quantitative diagnostic test such as procalcitonin. In this direction, we believe that the quantitative measurement of lung elasticity by sono-elastography (SE) could improve the diagnostic accuracy. 

Therefore, new non-invasive diagnostic options such as SE, currently used for other tissues (liver, breast, etc.), have been suggested to minimize the diagnostic bias generated by the isolated use of LUS over ALRTI consolidations. SE is a widely used medical imaging technique with multiple applications, the use of which began in 1990s. It provides information on tissue stiffness and can be used to complement conventional LUS findings, and adds a quantifiable and non-subjective value in the diagnosis and management of various ALRTI. Its main advantages include non-invasiveness, portability and low cost. All SE modalities are based on the assumption that soft tissues are more deformable than rigid tissues, so the stiffness characteristics of a lung tissue affected by ALRTI may differ depending on the type of condition [15]. 

This review aims to provide an in-depth overview of the technical fundamentals of SE, outlying its advantages compared to the conventional LUS and suggesting new opportunities for use in the management and assessment of children affected by ALRTI.

## 2. Materials and Methods

This review was completed by searching the Medline/Pubmed, Google Scholar and EMBASE databases for scientific publications, including original articles, meta-analyses, systematic reviews and clinical trials. Priority was given to the most relevant publications published during the last 10 years, particularly those relating to the paediatric population. The search keywords were: “pneumonia”, “lung ultrasound”, “elastography”, “sono-elastography”, “strain elastography” and “shear wave elastography”. After compiling and reviewing all publications by the research team, an updated description of the basic principles of ultrasound imaging and its main findings in relation to lung consolidation processes was carried out. Afterwards, the knowledge and description of the operation and applications of SE was deepened in order to finally propose a multidisciplinary SE image acquisition protocol, developed according to the literature reviewed, with special participation of the Diagnostic Imaging Unit and Pediatric Intensive Care teams from our institution. 

## 3. Lung Ultrasound: Where Do We Start

**Figure 1:**
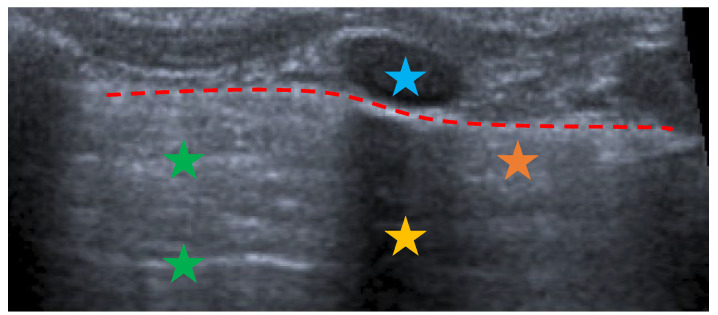
Ultrasound image corresponding to healthy lung tissue. The different structures and ultrasound artefacts can be seen: ribs (blue) causing a posterior acoustic shadow (yellow), pleural line (red), A-lines (green) and B-lines in normal amount (orange).

The patient bedside LUS is a widely used technique and it has been established as an essential alternative to chest X-ray for diagnosis of ALRTI. LUS has several advantages over chest X-ray, as it is faster, cheaper and does not emit radiation, so it allows the close monitoring of the evolution and the response to treatment [12,16,17,18]. From a physical point of view, LUS is based on the generation of variable frequency mechanical waves (2.5–20 MHz) towards the different tissues, where they are reflected, absorbed or scattered depending on the type of medium. It results in a 2D grayscale representation image provided by US devices. Normal lung parenchyma is made up of air, so the healthy lung image is in fact a compendium of artifacts that indicate the parenchyma is properly aerated (Figure 1). 

Linear or curved probes, depending on patient characteristics, are used to systemically scan six areas for each hemithorax (superior and inferior of each anterior, lateral and posterior zone) according to the international recommendations [19,20,21]. The evaluated findings are described as:

- The presence of A-lines.
- The presence of B-lines, their characteristics (short or long, spared or confluent) and their location (peri-lesional, monolateral/bilateral).
- The main lesion (consolidation): size, whether it is single or multiple, location (monolateral or bilateral). The presence of small subpleural consolidations (<1 cm).
- The presence of a bronchogram and its characteristics (air or fluid), morphology (branched or dot-like), dynamics during breathing (poorly or clearly dynamic); vascular pattern, presence of lung point and pulmonary pulse.
- The presence of lung sliding (M-mode).
- The presence and type of pleural effusion.

**Figure 2:**
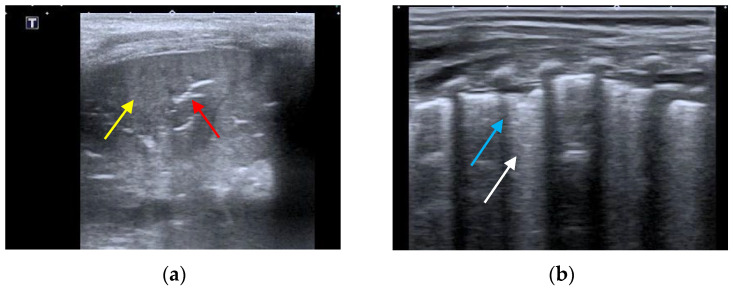
(**a**) BP seen by LUS: lung consolidation (yellow) with branching air bronchograms within it (red). (**b**) VP seen by LUS: coalescent B-lines (white) with small subpleural consolidations of less than 1 cm (blue), without bronchogram.

The different LUS patterns can be distinguished on the basis of the degree of air leakage and the presence of the different consolidation types based on previous publications [16,17,22,23]. BP pattern is based on the presence of lung consolidation with dynamic and branched air bronchogram within it and possible pleural line rupture (Figure 2a). VP pattern is based on the presence of diffuse or coalescent B lines with small, multiple and bilateral subpleural consolidations (Figure 2b) [12,17,24]. 

As a reminder, Table 1 highlights the main differences between chest X-ray and LUS in the diagnosis of different types of pneumonia.

**Figure 3:**
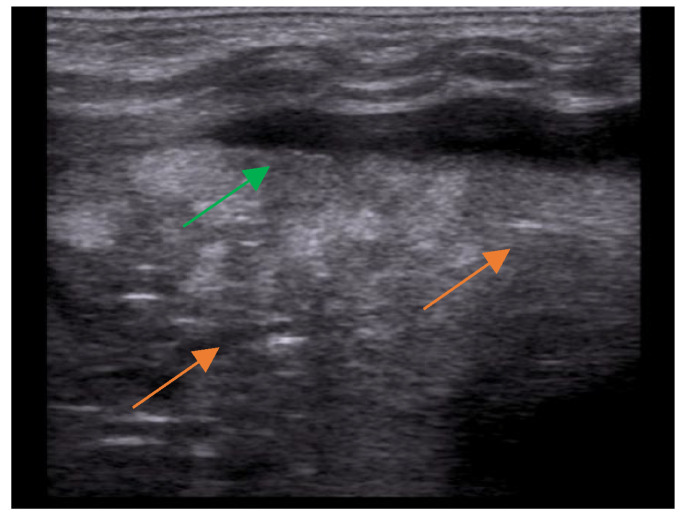
Atelectasis seen by LUS: lung consolidation (green) with parallel air bronchograms within it (orange).

One of the main differential diagnoses of pneumonia as a pulmonary consolidation is atelectasis, which can be identified using LUS by tissue-like consolidation with air (static or parallel) or fluid bronchogram within it (Figure 3). 

As mentioned above, and despite the different diagnostic LUS patterns described, sometimes it is still difficult to distinguish between the different entities of pulmonary condensation. For this reason, the use of new techniques such as lung SE has been suggested, allowing quantitative non-invasive measurements as additional information to support the final diagnosis of a specific type of lung consolidation in children. 

## 4. Ultrasound Elastography or Sono-Elastography

### 4.1. Fundamentals and Modalities of Sono-Elastography

SE is a new technique for assessing tissue stiffness. This property can be defined by Young’s modulus, which is defined as E = σ/ε, where σ is the applied stress and ε is the resulting deformation of the tissue. Therefore, while ultrasound analyses tissue acoustic properties, SE can provide additional tissue mechanical information [25,26]. 

**Figure 4:**
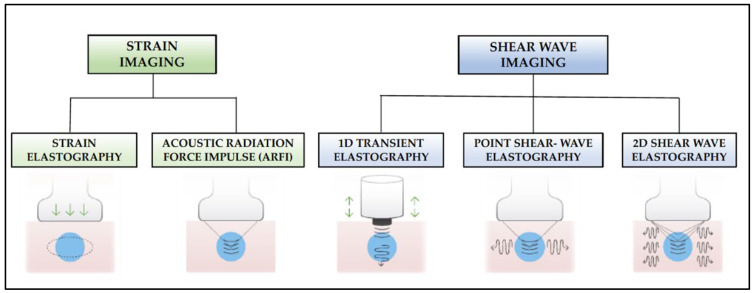
SE methods classified by data processing according to strain imaging or shear wave imaging. Figure partially adapted from [29].

SE techniques are based on the hypothesis that different pathological processes can lead to an alteration of normal tissue elasticity, as these techniques measure the degree of stiffness of a selected target tissue. It is also presumed that soft tissues suffer more deformation than hard tissues when they are submitted to an external force [27]. It is currently a widely used diagnostic test for the study of liver fibrosis, breast lesions, lymph nodes, etc., but its use for approaching lung conditions, especially in pediatrics, is still scarce. There are two main methods for SE measurements (Figure 4): semi-quantitative methods (strain imaging) and quantitative methods (shear-wave imaging). On the one hand, the first commercially available ultrasound (US) scanners to incorporate tissue stiffness assessment were based on the strain SE method, which estimates the qualitative relative strain of a target lesion in a region of interest (ROI) compared to that of the surrounding normal tissue [28]. The ratio of the strain measured in the normal tissue with that in the ROI, known as the strain ratio, provides information about the stiffness of the target lesion relative to the normal tissue. Thus, a strain ratio >1 indicates that the target lesion compresses less than the normal reference tissue (i.e., indicates greater stiffness) and vice versa.

There are two currently available strain SE modalities:

- **Strain elastography**: Depending on the type of stimulation, there are different strain elastography modalities. There are modes that require gentle compression with the transducer by the examiner, and other modes where pressure is generated by physiological movements of the patient, such as breathing. Whatever the mode of stimulation, there is a displacement of the tissue in the same direction as the pulse. This displacement (strain measurement) is recorded by the device as an indirect measure of tissue elasticity and plotted on a color map called an *elastogram*.
- **Acoustic Radiation Force Impulse (ARFI)**: Focused acoustic radiation pulses of short duration (0.1–0.5 ms) achieve tissue displacement in the same direction as the impulse, i.e., perpendicular to the skin surface. As a result, the generated waves are captured and displayed on a greyscale elasticity map, where the brightest areas correspond to those with the highest elasticity (soft tissues) and the darkest areas to those with the lowest elasticity (hard tissues). Thus, it does not require manual external compression and it provides a one-dimensional measure of tissue elasticity on a measurement area that can be positioned in a B-mode image plane.

**Figure 5:**
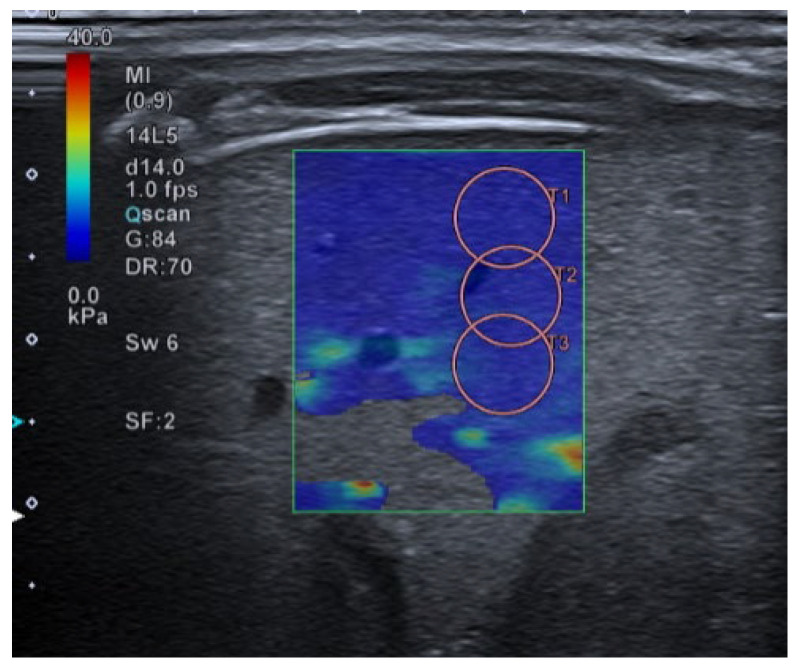
SWE applied on a healthy liver. The colour box in the ROI indicates the tissue is predominantly soft (blue), according to the device’s color chart.

On the other hand, the different shear-wave SE (SWE) modalities available on the new generation SE scanners use an external vibration or localised acoustic radiation force to displace the target tissue back and forth, parallel to the ultrasound stimulation. It creates a shear wave that propagates in a transverse direction from the initial stimulus. The result is a shear-wave front that sweeps both sides of the image plane from the initial point of application. The SWE device detects and measures, in real time, the propagation speed of this transverse shear wave, which is related to the elasticity of the tissue, so that the greater the stiffness of the tissue, the greater the speed of the wave. The quantitative value of the stiffness is obtained by converting the shear wave velocity (m/s) into Young’s modulus (kPa) using the relationship E = 3ρcs, where ρ is the tissue density and cs is the velocity [29,30]. The current shear-wave SE modalities are:

- **1D-Transient elastography (Fibroscan^TM^)**: This is based on the generation of an external vibration (50 Hz) that is transmitted from the body surface to the target tissue, where compression is produced. The speed of transmission of the resulting shear waves, which is proportional to the stiffness of the tissue, is then recorded (expressed in kPa). It is used mostly for the assessment of liver fibrosis in chronic liver disease (assessment of a tissue volume of 1 cm wide × 4 cm long). The advantages of this method is that it is fast and that it can be repeated throughout the patient’s follow-up. The main disadvantage is that, unlike other SE modalities, the measurement is not accompanied by a B-mode ultrasound image.
- **Point Shear Wave Elastography (pSWE)**: A pulse of acoustic radiation causes tissue displacement, in the normal direction and at a particular tissue location. However, the tissue displacement itself is not measured in this case. Instead, a portion of the longitudinal waves generated by the ARFI are converted into shear waves by the absorption of acoustic energy within the tissue. The shear wave velocity perpendicular to excitation plane is measured and used as a quantitative estimation of tissue elasticity. The higher the stiffness of the tissue, the higher the velocity of the resulting shear waves. In this modality, only a quantitative result is provided as no elasticity map is generated.
- **2D-Shear Wave Elastography (SWE)**: This is the latest and newest shear wave imaging technique. Like ARFI or pSWE, it uses acoustic radiation stimulation but, in this case, it rapidly scans multiple focal areas. This creates a virtual cylindrical shear wave cone that allows for the real-time monitoring of shear waves in 2D to measure their velocity, which is displayed on a quantitative colour map superimposed on a B-mode image (Figure 5). SWE has been extensively applied to characterize liver fibrosis [30,31], breast masses [32,33], prostate cancer lesions [34], thyroid nodules [35] and cervical lymph nodes [36]. In these contexts, SWE displayed low variability with respect to SE [37].

With this background, the aim of the present study is to assess whether transthoracic lung SWE may represent a new quantitative method suitable for the assessment of pulmonary consolidations.

### 4.2. What Role Does SE Play in the Study of Pulmonary Conditions?

As already mentioned, SE is a widely used technique for the study of the stiffness of certain organs such as the liver, breast lesions, thyroid, etc. Despite this, its use in the lung is still scarce and proof of this are the few studies that have been carried out in this respect, so there are no clear clinical indications for its use in the lung. Some research teams have worked on the approach to certain subpleural lesions or interstitial conditions by SE [38,39,40]. Quarato et al. [30] studied a total of 190 adult SWE patients with subpleural lesions and found no statistical differences in the shear wave velocities or their extrapolation to Young’s modulus between lung carcinomas, lung metastases or pneumonias. In a similar line of research, Ozgokce et al. [41] and Wei et al. [42] found different results for the sensitivity and specificity of diagnosing malignant subpleural lesions by SWE, although they selected different cut-off values. Lim et al. [27] evaluated a total of 45 patients with radiographic evidence of pneumonia, tumours or obstructive pneumonitis in 2016 to measure the strain ratio of different lesions. They found statistically significant differences between the strain ratios of necrosis, atelectasis, consolidations and tumours. 

In a recent publication, Koda et al. described an experimental lung model in which continuous shear wave elastography was used to determine the elasticity of lung tissue by measuring the shear wave phase difference between the B-lines observed by LUS [43]. The Mayo Clinic research group has published several research papers on the use of SE to assess interstitial lung pathology. They have developed a measurement method based on the application of a constant vibration to measure the wave velocity at three pulmonary points in each lung and they have found that there are different values in the fibrotic lung compared to healthy lung [44,45]. Despite the studies carried out, we strongly believe that there is a knowledge gap regarding the role of SE in the diagnosis of ALRTI, especially in children. We therefore hypothesize that the different ALRTI lead to regional alteration with respect to the basal elasticity of the lung tissue and that their characterization may be helpful in differentiating between infectious and non-infectious processes. In addition, it may also suggest the type of infection from which the patient is suffering. Therefore, we have developed a pulmonary image acquisition protocol using 2D-SWE in order to, in a second step, elucidate the usefulness or futility of this diagnostic technique in the management of children with ALRTI.

We hypothesize that lung consolidations have different stiffness than the baseline values of lung tissue, which has to be determined beforehand. The difference in stiffness between consolidations of infectious etiology and others of other causes, such as atelectasis, is still an uncertainty to be resolved by targeted clinical trials.

## 5. Lung SE Image Acquisition Protocol Proposal

After reviewing all the presented data, the authors suggest designing an image acquisition protocol to implement the use of lung SE and to analyze its results in order to improve and guide the etiological diagnosis of different lung consolidation processes. Following the scientific literature, an image acquisition protocol is proposed below.

**Figure 6:**
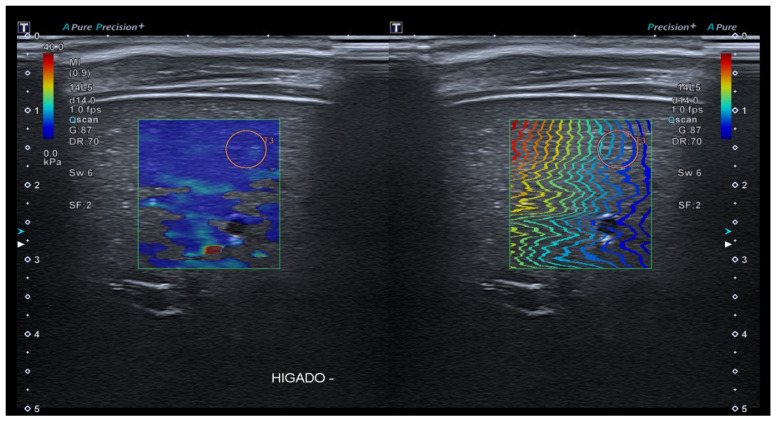
Application of SWE to liver tissue. The colour map (left) and the wave propagation map (right) are offered simultaneously while the device records all measurements.

**Figure 7:**
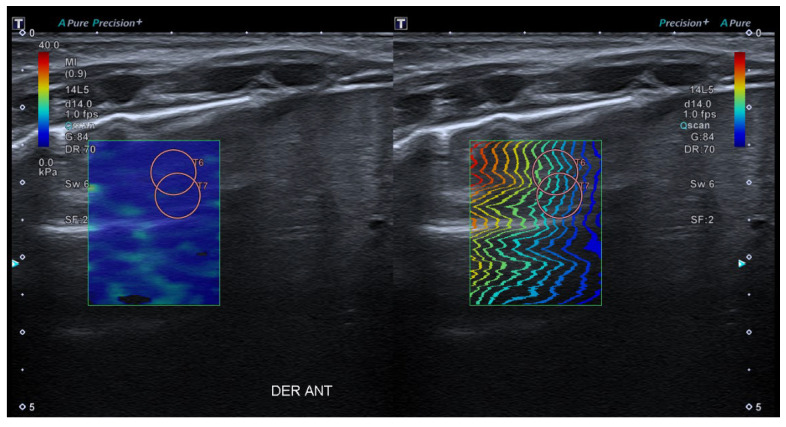
Application of SWE to pulmonary tissue. As in the liver, the colour map (left) and the wave propagation map (right) are offered simultaneously while the device records all measurements.

The standardization of the pulmonary image acquisition methodology, through an interdisciplinary protocol that homogenizes the data compilation, will reduce the variability of results by minimising the operator-dependent factor. The quantification of the lung SE values in patients without pulmonary pathology will provide normal values for this tissue. And, hypothetically, the quantification of the pulmonary SE values in patients with ALRTI or other pulmonary conditions will provide pathological values for different entities. The use of an ultrasound device with a high-resolution linear transducer with a frequency of 14 MHz is suggested (in our institution Canon^TM^ Aplio A500). The linear sub-preset within the abdomen preset should be used. The patient is examined in supine, prone, lateral decubitus, or sitting position, depending on his or her characteristics and age. As previously explained, the study of the organ’s elasticity is carried out using SWE imaging. Shear-wave speed (m/s) or its correlation to Young’s modulus should be acquired in liver tissue (gold standard) and in lung tissue (organ under study), to compare both results. The hepatic SWE should be carried out through the intercostal window (segment VII-VIII), using the linear preset with the transducer in a perpendicular position to the liver surface, horizontal to the liver capsule. (Figure 6). The pulmonary SE should be carried out through the intercostal window, using the linear preset with the transducer in a perpendicular position to the costal surface, horizontal to the pleura (Figure 7).

In both organs, SWE-*Real Time Shear Wave acquisition* mode is activated. A colored box is obtained 1 cm from the capsule and an elasticity scale is set at 40 kPa. The circular ROI with size number 5 should be selected: between 10 and 15 mm at a maximum depth of 5 cm. The inclusion of blood vessels and artefacts inside the ROI should be avoided. Between three and five acquisitions should be made, with only one ROI per acquisition. The mean value should be used, with the median IQR being less than 0.30. The values will be in KPa or cm/s. The authors suggest obtaining the SE values in the following order: liver, anterior right lung, anterior left lung, posterior right lung and posterior left lung.

## 6. Are There Other Elastography-Based Research Opportunities for Diagnosing Lung Processes?

Although the present manuscript focuses on the role of SE due to its greater availability for rapid use at the bedside, it is worth noting that several research groups have initiated non-invasive lung studies using elastography in combination with other tests such as MRI. MRI-elastography combines magnetic resonance imaging with low-frequency vibrations to produce a colour map that reflects tissue stiffness. Currently, MRI-elastography is mainly used to measure liver stiffness caused by fibrosis and inflammation in chronic liver disease. However, magnetic resonance elastography is also being investigated as a non-invasive diagnostic technique for the diagnosis of pathological processes in other parts of the body. Fakhouri et al. studied changes in shear stiffness in fibrotic pig lungs after the selective infusion of bleomycin. They found a progressive increase in stiffness over the weeks of the study (control stiffness of 1.02 ± 0.27 kPa at baseline, 0.68 ± 0.20 kPa in week 4 and 0.86 ± 0.29 kPa in week 8, whereas the stiffness of fibrotic lungs at baseline, in week 4 and week 8 were 0.98 ± 0.23, 1.52 ± 0.41, and 1.64 ± 0.40, respectively [46]).

Nevertheless, our team advocates the use of LUS and SE as first-line non-invasive diagnostic techniques because of their immediate availability at the bedside.

## 7. Conclusions

Lung SE is considered to be a new quantitative and non-invasive ultrasound measurement technique that could help in the etiological diagnosis of several pulmonary pathological processes. To this end, in addition to the homogenisation of elastographic image acquisition, using protocols such as the one presented in this paper, targeted clinical trials are needed to determine the normal values of lung elasticity, ideally in both children and adults. Secondly, it would be the basis of assessing whether pneumonia or other pulmonary pathological processes lead to a significant alteration of this elasticity. This would help in terms of diagnosis and treatment adjustment in those situations where the current diagnostic tests, in conjunction with clinical and analytical data, are not conclusive.

## Figures and Tables

**Table 1:** Main differences between chest X-ray and lung ultrasound for the diagnosis of pneumonia.

	Chest X-ray	Lung Ultrasound	
**Irradiation**	Yes	No	
**Bedside**	No	Yes	
**Reproducibility**	Not operator-dependent	Operator-dependent	
**Exploration areas**	Central and peripheral, with possibility of visualising the perihilar region	Peripheral, without possibility to visualise perihilar region	
**Characteristic imaging findings**	**Lobar pattern**: Unilateral consolidation of subsegmental, segmental, lobar or multilobar extension **Bronchopneumonic pattern**: bilateral and asymmetric patchy involvement **Interstitial pattern**: interlobular involvement with thickening of the interlobular septa and blurring of the vessel contours	**Bacterial pneumonia**	**Viral pneumonia**
		Tearing or thinning of pleural membrane Hypoechogenic tissue (larger than 1 cm) Arboriform air bronchogram (may or may not be dynamic) Pleural effusion Perilesional enhancement B-line	Small subpleural consolidations with an irregularly patched pattern Accumulation of perilesional B-lines

## Data Availability

All the data are contained within the published version of the manuscript.
